# Repositioning drugs for inflammatory disease – fishing for new anti-inflammatory agents

**DOI:** 10.1242/dmm.016873

**Published:** 2014-07-18

**Authors:** Christopher J. Hall, Sophie M. Wicker, An-Tzu Chien, Alisha Tromp, Lisa M. Lawrence, Xueying Sun, Geoffrey W. Krissansen, Kathryn E. Crosier, Philip S. Crosier

**Affiliations:** Department of Molecular Medicine and Pathology, School of Medical Sciences, University of Auckland, Auckland 1023, New Zealand

**Keywords:** Drug repositioning, Zebrafish, Inflammation, Neutrophil, Atopic dermatitis, Immunity

## Abstract

Inflammation is an important and appropriate host response to infection or injury. However, dysregulation of this response, with resulting persistent or inappropriate inflammation, underlies a broad range of pathological processes, from inflammatory dermatoses to type 2 diabetes and cancer. As such, identifying new drugs to suppress inflammation is an area of intense interest. Despite notable successes, there still exists an unmet need for new effective therapeutic approaches to treat inflammation. Traditional drug discovery, including structure-based drug design, have largely fallen short of satisfying this unmet need. With faster development times and reduced safety and pharmacokinetic uncertainty, drug repositioning – the process of finding new uses for existing drugs – is emerging as an alternative strategy to traditional drug design that promises an improved risk-reward trade-off. Using a zebrafish *in vivo* neutrophil migration assay, we undertook a drug repositioning screen to identify unknown anti-inflammatory activities for known drugs. By interrogating a library of 1280 approved drugs for their ability to suppress the recruitment of neutrophils to tail fin injury, we identified a number of drugs with significant anti-inflammatory activity that have not previously been characterized as general anti-inflammatories. Importantly, we reveal that the ten most potent repositioned drugs from our zebrafish screen displayed conserved anti-inflammatory activity in a mouse model of skin inflammation (atopic dermatitis). This study provides compelling evidence that exploiting the zebrafish as an *in vivo* drug repositioning platform holds promise as a strategy to reveal new anti-inflammatory activities for existing drugs.

## INTRODUCTION

The inflammatory response is a complex reflexive process that protects the host against infection and injury to maintain homeostasis (Medzhitov, 2008). Collectively, inflammatory disorders including autoimmune diseases, allergies, asthma and sepsis are a major cause of illness and death. It is also becoming apparent that low-grade chronic inflammation underlies many diseases, including type 2 diabetes, cancer, cardiovascular disease and neurodegeneration, that previously were not considered to possess a strong inflammatory component (Mathis and Shoelson, 2011). These studies are redefining how we view inflammation and revealing new pathways that could be targeted therapeutically. As such, there is strong interest in identifying new anti-inflammatory drugs to augment or replace current therapies (Ward, 2008). Current strategies to treat inflammation largely rely on corticosteroids and non-steroidal anti-inflammatory drugs (NSAIDs), which have well-documented side effects, including osteoporosis, impaired wound healing, ulcerogenic effects and stroke (O’Neill, 2006; Ward, 2008). Despite the success of more contemporary biopharmaceuticals, including tumor necrosis factor-α (TNF-α) inhibitors, there still exists an unmet need for new anti-inflammatory drugs (O’Neill, 2006; Ward, 2008).

Traditional *de novo* drug discovery approaches have largely failed to deliver on promises of improved productivity, despite large increases in funding (Ashburn and Thor, 2004). This has led pharmaceutical and biotech companies to explore new strategies to improve productivity. One such strategy is drug repositioning (also known as repurposing or reprofiling). Drug repositioning is the process of identifying new uses for drugs outside the scope of their original medical indication. By exploiting existing knowledge of drugs, drug repositioning can offer a faster and cheaper approach than traditional drug discovery. Drug repositioning has become an increasingly important part of the drug development landscape, with many pharmaceutical and biotech companies now having repositioning programs (Arrowsmith and Harrison, 2012). The philosophy of drug repositioning is underpinned by the emerging realization that common molecular pathways are often shared among seemingly diverse diseases. Therefore, drugs originally identified as efficacious in one disease could potentially be of therapeutic benefit in another. With lower costs, shorter development times and higher success rates, drug repositioning is also ideally suited for academia-based drug discovery (Oprea et al., 2011).

Zebrafish are emerging as a valuable drug discovery platform. Zebrafish embryos and larvae permit a live whole vertebrate bioassay approach to define and characterize drug activity in a high-content fashion. Micromolar quantities of drug can be administered to embryos by simple immersion and wash-out protocols, providing a cost-effective alternative to expensive mammalian approaches with the added benefit of precise temporal control of drug delivery and exposure time (Zon and Peterson, 2005; Kaufman et al., 2009; Bowman and Zon, 2010; Taylor et al., 2010). Zebrafish can also offer an informative intermediate triaging step between cell-based *in vitro* studies and more time-intensive/expensive mammalian platforms for assessing the effects of drugs. Highlighting the success of chemical-genetic screening in zebrafish, compounds have moved from zebrafish screens to Phase 1b clinical trials in less than 5 years (North et al., 2007; Goessling et al., 2011; Martz, 2011).

The zebrafish is a well-established model in which to study leukocyte behavior. By 2 days post-fertilization (dpf), zebrafish embryos are populated with neutrophil and macrophage lineages that function with remarkable similarity to those in humans. Exploiting the transparency of zebrafish embryos and early larvae, live imaging within neutrophil- and macrophage-lineage-specific transgenic reporter lines has given researchers access to explore the function of these cells, in real time, within a completely intact animal model. When combined with the genetic tractability afforded by this system, unique insights into their function during different pathological conditions have been revealed (Mathias et al., 2006; Renshaw et al., 2006; Hall et al., 2007; Niethammer et al., 2009; Ellett et al., 2011; Yoo et al., 2011; Hall et al., 2012; Pase et al., 2012; Yang et al., 2012; Hall et al., 2013; Roca and Ramakrishnan, 2013). This model has also given new insights into the inflammatory response that is superimposed on the wound healing process (Mathias et al., 2006; Niethammer et al., 2009; Yoo et al., 2011; Pase et al., 2012). Similar to mammals, neutrophils are the first leukocytes to migrate to wounded tissues, where their numbers peak prior to those of macrophages, which arrive slightly later and persist for longer (Martin and Leibovich, 2005; Ellett et al., 2011; Gray et al., 2011). Neutrophilic inflammation then resolves through a combination of apoptosis and reverse migration (Mathias et al., 2006; Loynes et al., 2010; Starnes and Huttenlocher, 2012). Recently, chemical-genetic screening in zebrafish has been coupled with live imaging of neutrophil behavior to identify chemical modulators of the neutrophil inflammatory response (Robertson et al., 2014; Wang et al., 2014). With a view to identifying new anti-inflammatory drugs, tanshinone IIA (derived from a Chinese medicinal herb) has been shown to accelerate resolution of neutrophilic inflammation (Robertson et al., 2014). In a similar study, sterigmatocystin and antibiotic PF1052 (identified from a natural compound library) were effective inhibitors of neutrophil migration (Wang et al., 2014). Importantly, the effects of these drugs were demonstrated to be conserved *in vitro* using mammalian neutrophils.

**TRANSLATIONAL IMPACT**

**Clinical issue**

Inflammation is a physiological response of host tissues to infection and injury. However, dysregulation of this response can contribute to a broad range of diseases, ranging from inflammatory dermatoses to type 2 diabetes and cancer. The mainstays of treatment for inflammation are the non-steroidal and corticosteroid classes of anti-inflammatory drugs. Although widely used, these compounds have several adverse effects. For example, when used to treat inflammatory skin conditions, such as atopic dermatitis (AD), they can cause hypopigmentation, striae (stretch marks on the skin), telangiectasia (small dilated vessels) and thinning of the skin. Therefore, new therapeutic options are needed to treat inflammation. Drug repositioning is the process of finding new uses for existing drugs and thus represents a faster and cheaper alternative to traditional drug discovery. Because molecular pathways are often shared among seemingly diverse diseases, drugs that originally proved therapeutic in one disease could also be effective in another. Because of the complexity of the inflammatory response, the identification of new therapeutic compounds for inflammatory conditions is especially suited to repositioning approaches.

**Results**

The zebrafish is an established drug discovery platform that is particularly suitable for *in vivo* phenotypic drug screening. In this study, the authors exploited this model to test the repositioning of drugs for inflammatory diseases. They first used the tail fin wounding assay, an acute model of inflammation, which involves the amputation of the zebrafish tail fin and enables live imaging of neutrophil immune cells that are recruited during inflammation. Via a drug screening protocol, the authors identified a number of drugs (repositioned drugs) with the ability to suppress neutrophil recruitment that had not previously been characterized as general anti-inflammatory agents. In addition, using an ovalbumin-mediated epicutaneous mouse model of AD, the authors showed that several of these repositioned drugs (which include amodiaquin dihydrochloride, alfuzosin hydrochloride and clonidine hydrochloride) can significantly suppress dermatitis-related inflammation.

**Implications and future directions**

This study highlights the utility of the zebrafish system as an *in vivo* drug discovery platform to reposition existing drugs for anti-inflammatory applications. This study also revealed several repositioned drugs that might provide alternative therapeutic options to suppress inflammation associated with AD. The repositioning approach is therefore particularly useful to speed up the discovery of new drugs with potential therapeutic effects for human inflammatory diseases.

We attempted to exploit the zebrafish as a drug repositioning platform to help reveal new anti-inflammatory activities for existing drugs. Utilizing a fin amputation model of acute inflammation, we interrogated known drugs (selected for their chemical and pharmacological diversity) to identify those that suppress neutrophil migration. We identified a large number of drugs that significantly impaired neutrophil recruitment, as detected within the neutrophil-specific *Tg(lyz:EGFP)* reporter line (Hall et al., 2007). Ranking hits by significance of effect and removing known anti-inflammatories (NSAIDs and corticosteroids), we selected the ten most significant inhibitors that we called our repositioned drug set. Live imaging of the effects of these drugs on neutrophil migration to wounded tissue revealed that most drugs affected migration velocity. Importantly, we show the anti-inflammatory activities of these repositioned drugs translated to an *in vivo* epicutaneous sensitization mouse model of atopic dermatitis (AD), an inflammatory skin disease. These results highlight the utility of the zebrafish system as a drug repositioning platform, in particular its potential to repurpose existing drugs for new anti-inflammatory uses.

## RESULTS

### Establishing an *in vivo* screening platform to identify new anti-inflammatory activities for existing drugs

**Fig. 1.**
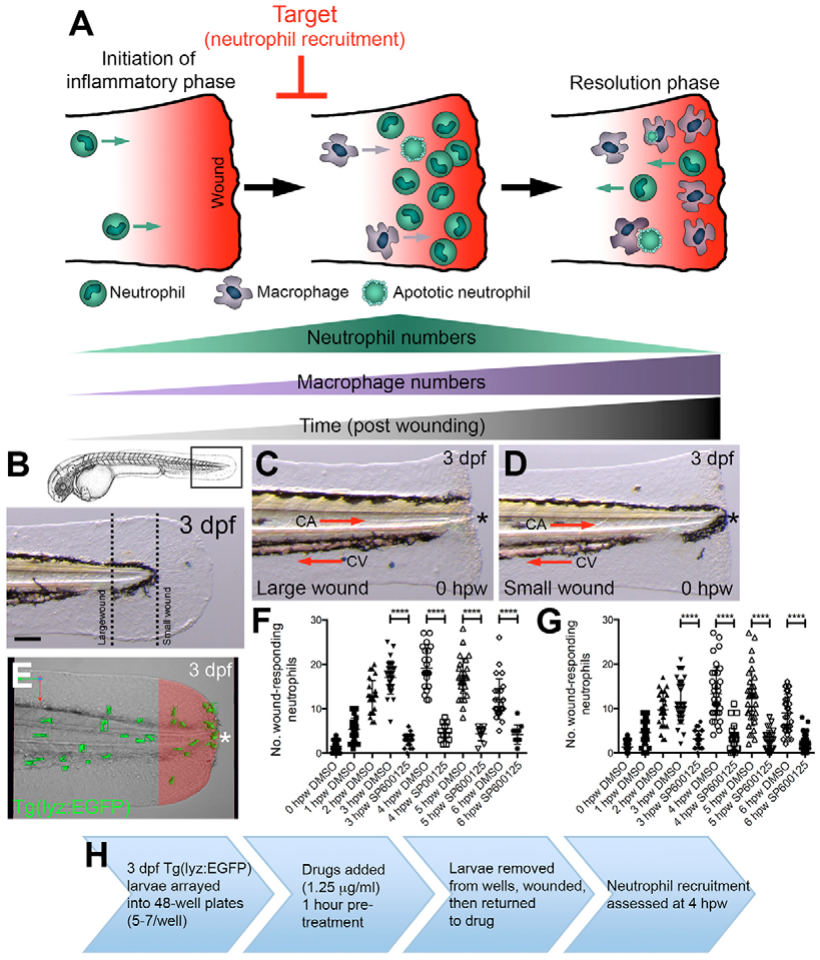
**Model of inflammatory response during tail fin wounding and work flow for neutrophil-recruitment anti-inflammatory screen.** (A) Schematic illustrating recruitment and fate of neutrophils during larval tail fin wounding. (B) Anatomical locations of sterile tail transections to generate ‘large’ and ‘small’ tail fin wounds within 3-dpf larvae. Scale bar: 50 μm. (C,D) Examples of ‘large’ and ‘small’ wounds, respectively, immediately following transection (0 hpw). CV, caudal vein; CA, caudal artery. All views anterior to left. Asterisks mark wound border. (E) Live imaging of the recruitment of neutrophils within 3-dpf *Tg(lyz:EGFP)* larvae following tail fin wounding. A wound region (100 μm from wound border) is highlighted in red. Red and green arrows indicate dorsal-ventral and anterior-posterior axes, respectively. (F,G) Quantification of wound-responding neutrophils (number of neutrophils within the wound region, as shown in E) following ‘large’ and ‘small’ wounds, as detected at 0, 1, 2, 3, 4, 5 and 6 hpw. ****P<0.0001. (H) Schematic illustrating work flow for neutrophil-recruitment anti-inflammatory screen.

The zebrafish model has been used extensively to further understand leukocyte function during inflammation using tail fin wounding assays (Mathias et al., 2006; Renshaw et al., 2006; Niethammer et al., 2009; Ellett et al., 2011; Gray et al., 2011; Yoo et al., 2011; Pase et al., 2012). Wound healing relies on a series of complex interactions between soluble mediators, extracellular proteases, resident cells and infiltrating leukocytes (Eming et al., 2007). Broadly speaking, wound healing can be divided into three overlapping phases: inflammation, tissue formation and tissue remodeling. Neutrophil recruitment is one of the earliest events marking the initiation of the inflammatory phase. Imaging of neutrophil behavior in living zebrafish embryos and larvae has revealed that, much like mammals, neutrophil numbers peak within inflamed tissues before that of macrophages, which arrive slightly later to help remove apoptotic neutrophil debris as part of the resolution phase of inflammation (Ellett et al., 2011; Gray et al., 2011). Superimposed on these events, reverse neutrophil migration, a phenomenon first observed in the zebrafish model system, has been shown to contribute to the resolution of neutrophilic inflammation (Mathias et al., 2006; Hall et al., 2007; Starnes and Huttenlocher, 2012) (Fig. 1A). A large number of pathways orchestrate leukocyte migration during inflammation (and hence there is a large number of potential pathways that a drug could interfere with); therefore, our strategy was to focus on the narrow temporal window during which peak numbers of neutrophils accumulate in response to tail fin amputation (Fig. 1A).

We have previously shown that the migratory response of fluorescent neutrophils towards wounds, as marked within *Tg(lyz:EGFP)* larvae, is proportional to the size of the wound (Hall et al., 2009a). In light of this, we first wanted to establish a scalable tail fin amputation protocol that generated a robust and reproducible inflammatory response, as measured by the recruitment of fluorescent neutrophils within the *Tg(lyz:EGFP)* reporter line. We identified a pigmentation pattern within the caudal part of the tail of 3-dpf larvae that provided an anatomical landmark where tail fins could be amputated to generate ‘large’ wounds of similar size (Fig. 1B,C). Another anatomical landmark was provided by the posterior-most extremity of the notochord, where fin amputation created a ‘small’ wound (Fig. 1B,D). When quantifying the numbers of wound-responding neutrophils [defined as the number of neutrophils within 100 μm of the wound border (Fig. 1E)], in addition to recruiting more neutrophils, ‘large’ wounds possessed less inter-wound variation, when compared with ‘small’ wounds. Of note, both wounds possessed the same temporal profile, with numbers peaking at 4 hours post-wounding (hpw) (Fig. 1F,G). Treatment with the JNK inhibitor SP600125, which has previously been shown to suppress the recruitment of neutrophils to tail fin wounds similar to those in this study, inhibited neutrophil recruitment at all time points examined (Zhang et al., 2008; Robertson et al., 2014) (Fig. 1F,G). Of note, a 1-hour pre-treatment period was necessary for this drug-mediated suppression of neutrophil recruitment, as previously reported (Zhang et al., 2008) (data not shown).

In light of the above results, we designed a screening assay where 3-dpf *Tg(lyz:EGFP)* larvae were arrayed into 48-well plates (5–7 larvae/well) and initially exposed to a 1-hour drug pre-treatment followed by fin amputation (where a ‘large’ wound was generated) prior to being returned to drug. Larvae were then fixed at 4 hpw for immunofluorescence detection of GFP and subsequent quantification of wound-responding neutrophils, as detected within an arbitrarily defined 100-μm wound region (Fig. 1H). Each 48-well plate contained two DMSO and two SP600125 treatments, which served as negative and positive controls, respectively. We selected the Prestwick Chemical Library^®^, which contains 1280 drugs with high chemical and pharmacological diversity. Possessing 100% approved drugs, this library is designed to reduce low-quality hits and is ideally suited to repositioning-focused screening. In a preliminary pilot screen using a random collection of test drugs, a concentration of 5 μg/ml resulted in a small proportion of treated larvae possessing mild cardiac edemas and labored circulation. Owing to the importance of a functioning cardiovascular system for the inflammatory response, we used a concentration of 1.25 μg/ml, which resulted in no detectable effect on the cardiovascular system (data not shown).

### A zebrafish drug repositioning screen identifies previously unknown anti-inflammatory activities for drugs

**Fig. 2.**
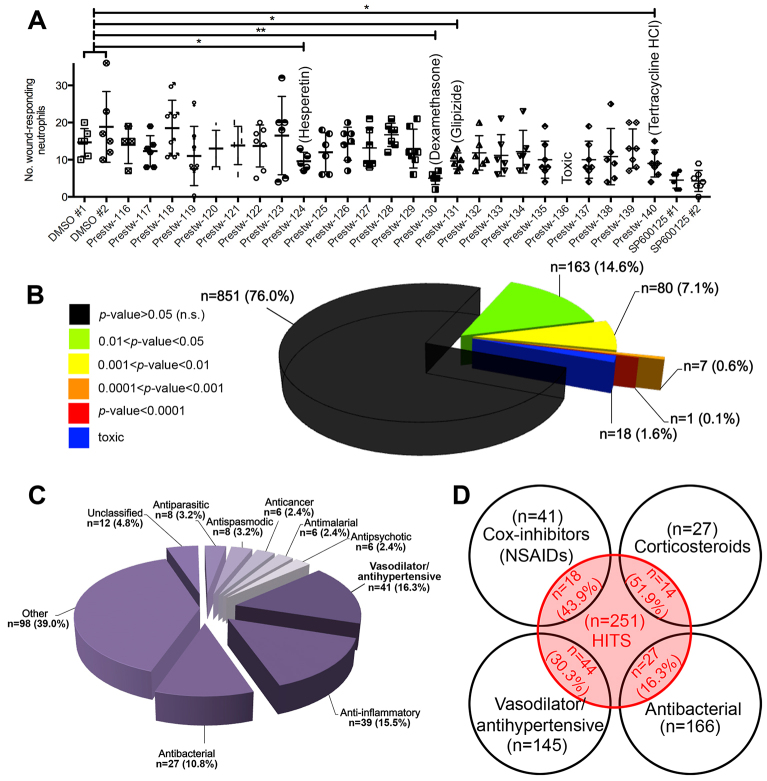
**Hits from the neutrophil-recruitment anti-inflammatory screen.** (A) Example read-out from the neutrophil-recruitment anti-inflammatory screen revealing the hits hesperetin (flavanoid), dexamethasone (corticosteroid), glipizide (antidiabetic) and tetracycline HCl (antibacterial). *P<0.05; **P<0.01. (B) Hits categorized by significance of effect on neutrophil recruitment, when compared with DMSO (−ve) controls. (C) Hits categorized by therapeutic group. The category ‘Other’ represents therapeutic groups containing <six hits. (D) The numbers and proportion of cox inhibitors (NSAIDs), corticosteroids, vasodilator/antihypertensive and antibacterial drugs screened that demonstrated a significant effect on neutrophil recruitment.

Our screening and quantification approach enabled the ranking of hits, with respect to their significance on neutrophil recruitment, when compared to negative (DMSO-treated) controls. An average *P*-value was calculated (using unpaired, two-tailed *t*-tests) by comparing the effect of a specific drug against the two DMSO controls on the same plate (Fig. 2A). This analysis revealed that 251 (22.4%) screened drugs had a significant effect on neutrophil recruitment (*P*<0.05) (Fig. 2B). Of note, at the tested concentration, 18 (1.6%) screened drugs induced toxic effects (defined as any observable effect on the cardiovascular system or other gross morphological defects). By grouping hits based on significance, 1 (0.1%), 7 (0.6%), 80 (7.1%) and 163 (14.6%) screened drugs demonstrated *P*<0.0001, 0.0001<*P*<0.001, 0.001<*P*<0.01 and 0.01<*P*<0.05, respectively (Fig. 2B). Analysis of the therapeutic groups represented by the 251 hits revealed that vasodilators/antihypertensives (*n*=41, 16.3%), anti-inflammatories (*n*=39, 15.5%) and antibacterials (*n*=27, 10.8%) were the most represented drug classes (Fig. 2C). Importantly, 43.9% of all NSAIDs and 51.9% of all corticosteroids present in the library were identified as hits in our screen, validating our screening system and supporting the future translation of our hits into a mammalian setting (Fig. 2D).

For subsequent validation and translation experiments, we decided to focus on the ten most significant hits that had not previously been characterized as general anti-inflammatory agents. Following removal of the NSAIDs etodolac and niflumic acid from the 12 most significant hits, the following ten drugs remained: chlorphensin carbamate (muscle relaxant), fludrocortisone acetate (treatment of Addison’s disease), acetohexamide (antidiabetic), methyldopa (L,-) (antihypertensive), pinacidil (vasodilator), amodiaquin dihydrochloride (antimalarial), alfuzosin hydrochloride (antihypertensive), mafenide hydrochloride (antibacterial), nefopam hydrochloride (analgesic) and clonidine hydrochloride (antihypertensive) (Table 1). Hereafter we refer to this group as our repositioned drug set.

**Table 1.**
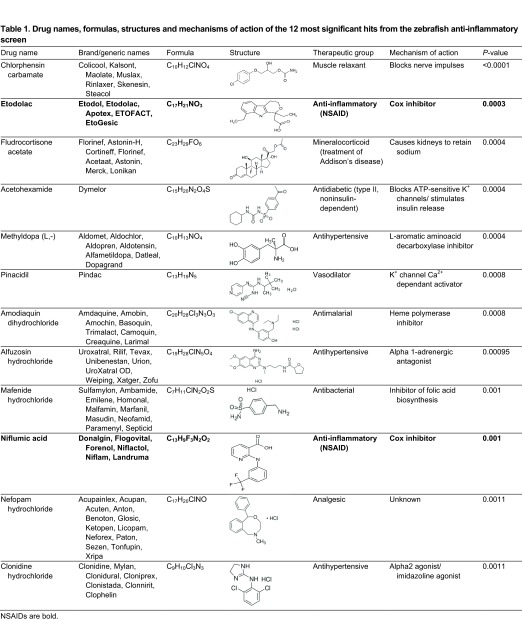
Drug names, formulas, structures and mechanisms of action of the 12 most significant hits from the zebrafish anti-inflammatory screen

### Repositioned drugs suppress neutrophilic inflammation in a dose-dependent fashion

**Fig. 3.**
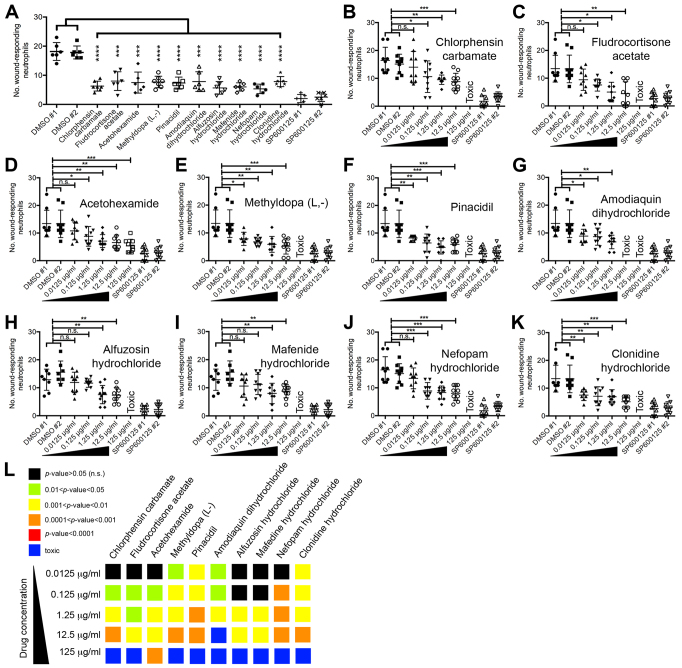
**Validation of repositioned drug set with newly sourced drug and assessment of dose response.** (A) Secondary screen validation of repositioned drug set using newly sourced drug. (B–K) Assessing the dose response of chlorphensin carbamate (B), fludrocortisone acetate (C), acetohexamide (D), methyldopa (L,-) (E), pinacidil (F), amodiaquin dihydrochloride (G), alfuzosin hydrochloride (H), mafenide hydrochloride (I), nefopam hydrochloride (J) and clonidine hydrochloride (K) using the neutrophil-recruitment anti-inflammatory assay. (L) Summary of dose responses of repositioned drug set presented as a heat map, where warmer colors represent a more significant effect on neutrophil recruitment, when compared with DMSO-treated controls. Abbreviations: n.s., not significant; * *P*<0.05; **P<0.01; ***P<0.001; ****P<0.0001.

When performing zebrafish chemical genetic screens it is necessary to follow up hits from primary screens by repeating in a secondary assay with alternatively sourced drug and perform a dose response (Kaufman et al., 2009). We sourced larger quantities of our selected hits and validated their effect on neutrophil recruitment following fin amputation (Fig. 3A). We then performed a dose response from 0.0125–125 μg/ml. All drugs increasingly suppressed neutrophil recruitment to wounds with higher concentration (Fig. 3B–L). Of note, with the exception of acetohexamide, all drugs induced toxic effects at the highest concentration tested (125 μg/ml). To eliminate the possibility that our observed drug-induced decreases in wound-responding neutrophils were secondary to an ablative effect on neutrophils, we assessed whole larvae neutrophil numbers by flow cytometry following a 5-hour drug treatment (at the 1.25 μg/ml screening dose). This analysis revealed that none of the repositioned drugs significantly affected whole larvae numbers of neutrophils (see supplementary material Fig. S1). These results confirm that these drugs (our repositioned drug set) suppress neutrophil recruitment to wounds in a dose-dependent fashion.

### Live imaging of the effects of repositioned drugs on the neutrophil response to tissue injury reveals that drugs largely affect migration velocity and displacement rate

**Fig. 4.**
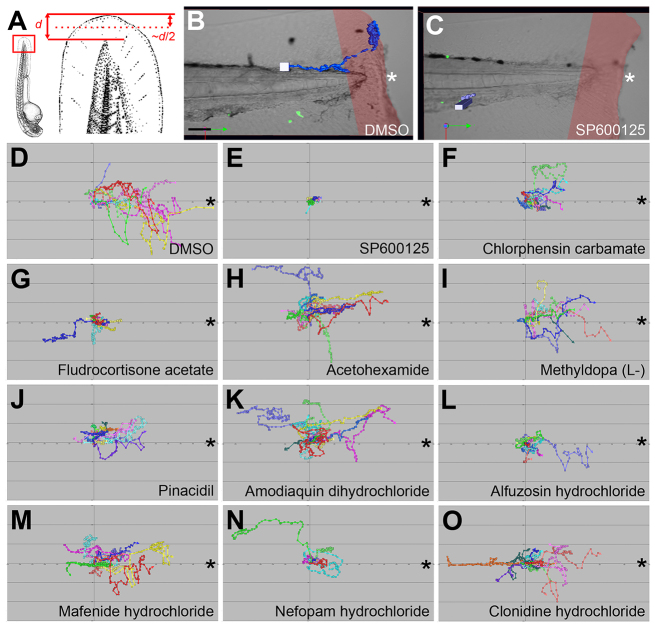
**Repositioned drug set disrupts neutrophil migration to tail fin wounds.** (A) Schematic illustrating the location of tail fin amputations used for live imaging studies. *d*, distance. (B,C) Examples of tracked neutrophils in wounded 3-dpf *Tg(lyz:EGFP)* larvae treated with DMSO or SP600125, respectively. Red shading indicates wound region. (D–O) Individual neutrophil tracks (from two individual larvae) in wounded 3-dpf *Tg(lyz:EGFP)* larvae treated with DMSO (D), SP600125 (E), chlorphensin carbamate (F), fludrocortisone acetate (G), acetohexamide (H), methyldopa (L,-) (I), pinacidil (J), amodiaquin dihydrochloride (K), alfuzosin hydrochloride (L), mafenide hydrochloride (M), nefopam hydrochloride (N) and clonidine hydrochloride (O). All views anterior to left. Asterisks mark wound border. Scale bar: 50 μm in B.

Live imaging of wounded transgenic zebrafish larvae possessing fluorescent neutrophils has provided unique insights into the migratory kinetics of these innate immune cells (Niethammer et al., 2009; Yoo et al., 2011; Pase et al., 2012). We live-imaged the effect of our repositioned drug set on neutrophil migration kinetics within 3-dpf *Tg(lyz:EGFP)* larvae from ~1–4 hpw. Of note, when generating wounds for live-imaging experiments, smaller wounds [resulting from fin amputations halfway between the posterior-most extremity of the notochord and the posterior fin edge (Fig. 4A)] were generated. This was to create a smaller neutrophil response, making it easier to track individual neutrophils. This analysis confirmed that fewer neutrophils reached the wound region in the presence of the repositioned drugs, when compared with DMSO-treated controls (Fig. 4B–O).

**Fig. 5.**
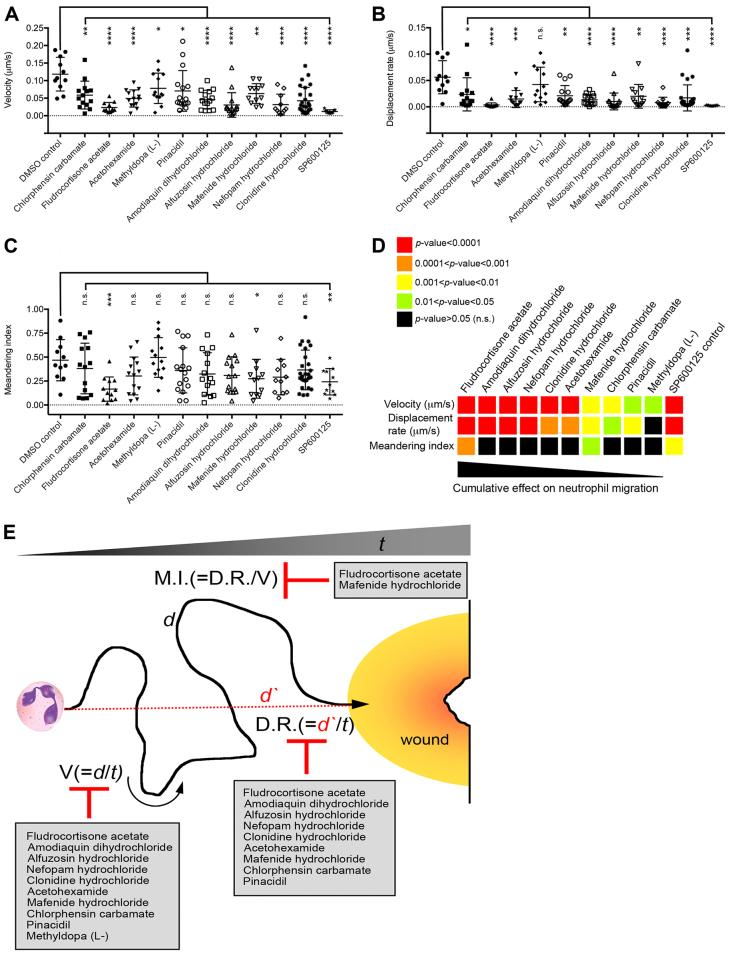
**Repositioned drug set disrupts specific aspects of neutrophil migration to tail fin wounds.** (A–C) The effects of selected drugs on neutrophil velocity (A), displacement rate (B) and meandering index (C) when tracked in 3-dpf *Tg(lyz:EGFP)* wounded larvae, relative to DMSO controls [neutrophil tracks generated from two individual experiments (wounded larvae)/treatment]. n.s., not significant; *P<0.05; **P<0.01; ***P<0.001; ****P<0.0001. (D) Summary of effects of repositioned drug set on velocity, displacement rate and meandering index presented as a heat map, where warmer colors represent a more significant effect, when compared with DMSO-treated controls. The drugs are ordered from left to right with respect to their cumulative effect on neutrophil migration from the live-imaging analysis. (E) Schematic grouping of selected drugs by their ability to influence specific aspects of neutrophil migration. M.I., meandering index; D.R., displacement rate; V, velocity; *t*, time; *d*, distance; *d*′, straight line distance.

Further analysis of these neutrophil tracks revealed that most drugs affected migration velocity (speed of neutrophil migration over track) and displacement rate (straight-line distance from track start to end divided by track time) (Fig. 5A,B). In contrast, only fludrocortisone acetate and mafenide hydrochloride affected the meandering index, which provides a measure of a track’s deviation from a straight line (Fig. 5C). When ordering the repositioned drug set with respect to their cumulative effects on these neutrophil migration parameters (based on significance of effect when compared with DMSO-treated controls), the drug set appeared to cluster into two distinct groups (Fig. 5D). Fludrocortisone acetate, amodiaquin dihydrochloride, alfuzosin hydrochloride, nefopam hydrochloride, clonidine hydrochloride and acetohexamide affected neutrophil migration kinetics more significantly than mafenide hydrochloride, chlorphensin carbamate, pinacidil and methyldopa (L,-) (Fig. 5D,E).

### The anti-inflammatory activities of repositioned drugs translate to a mouse model of skin inflammation

**Fig. 6.**
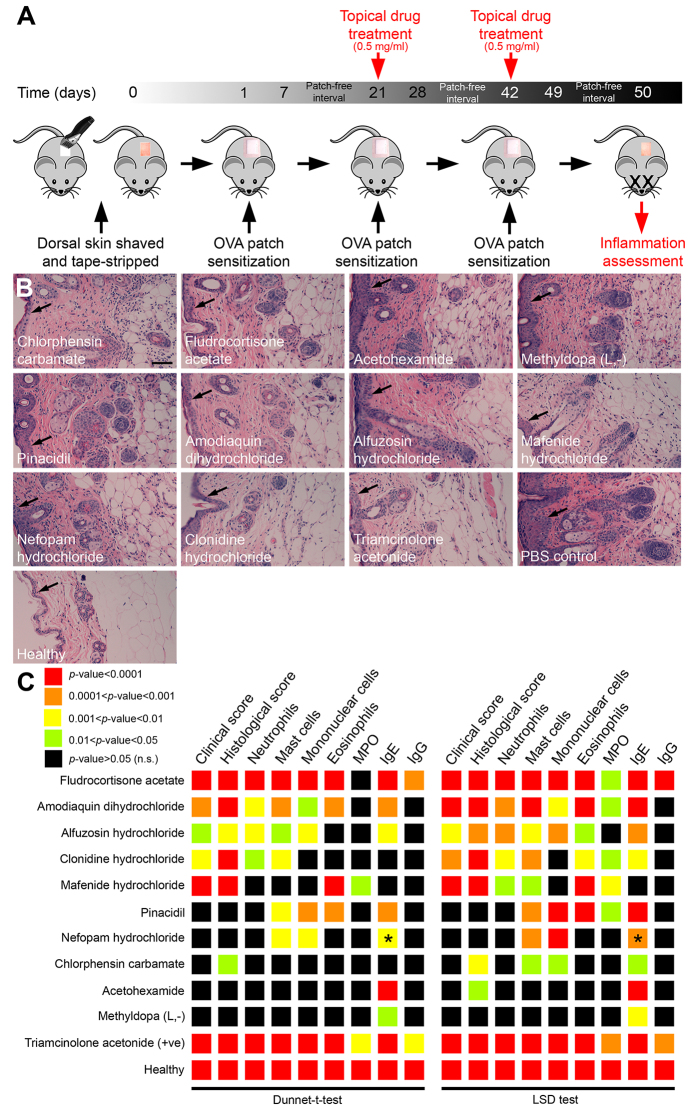
**Repositioned drug set demonstrates anti-inflammatory activity in an ovalbumin-mediated epicutaneous sensitization model of AD.** (A) Schematic illustrating the design of ovalbumin-mediated epicutaneous sensitization model of AD and drug delivery strategy. Shaved exposed skin was tape-stripped prior to sensitization with the allergen ovalbumin (OVA) (applied via a patch of sterile gauze secured with bio-occlusive dressing). Mice received three individual OVA exposures. On the second and third sensitizations, sterile drug (at 0.5 mg/ml dose) was applied (or sterile PBS as a negative control). Mice were anesthetized 1 day following removal of the final gauze patch and blood/skin samples collected for assessment of inflammation. (B) Hematoxylin- and eosin-stained skin sections following drug treatment, compared with PBS-treated and healthy controls. Arrows mark epidermis. Scale bar: 100 μm. (C) Summary of effects of selected drugs on clinical score (visual assessment), histological score (examination of dermis and hypodermis from hematoxylin- and eosin-stained skin sections), histological quantification of neutrophils, mast cells, mononuclear cells and eosinophils, and ELISA quantification of MPO and OVA-specific IgE and IgG. The anti-inflammatory effects of each drug [with respect to the different inflammation parameters and relative to PBS (−ve) controls] are presented as a heat map, where warmer colors represent a more significant reduction in the specific parameter. Asterisks mark significant increases in an inflammation parameter, relative to PBS controls.

Characterized by chronically relapsing cutaneous inflammation, AD is an increasingly common allergic skin disease (Boguniewicz and Leung, 2011). Strategies to treat inflammatory dermatoses largely rely on the immunosuppressive action of corticosteroids. In addition to beneficial effects, including limiting the recruitment and/or activation of immune cells (such as neutrophils), corticosteroids negatively impact the ability of the skin to repair, suggesting that alternative therapeutic approaches to alleviate skin inflammation could be of benefit (Guillot, 2013). We decided to investigate conservation of activity of our repositioned drug set in an *in vivo* mouse model of AD (an inflammatory skin disease) for the following reasons: (1) our zebrafish tail fin wounding assay represented a simple model of skin inflammation; (2) the ovalbumin-mediated epicutaneous model of AD enables evaluation of multiple parameters associated with inflammation (Sun et al., 2011); and (3) current therapeutic treatments for AD are limited. Of note, a previous study had shown a therapeutic effect for fludrocortisone acetate in treating allergic dermatitis (Vollmer, 1956). We decided to include this drug in our mammalian translation experiments as a further positive control. We selected an epicutaneous sensitization model of AD that closely resembles the pathophysiology of AD in humans (Jin et al., 2009). In brief, the dorsal skin of mice is shaved and tape-stripped several times to mimic skin injury inflicted in AD patients by skin scratching. The allergen ovalbumin (OVA) is then administered (via bio-occlusive dressing) by three separate 1-week exposures separated by 2-week patch-free intervals. Mice are then sacrificed at 50 weeks to assess inflammation using a set of measurable inflammatory parameters (Jin et al., 2009; Sun et al., 2011). Our strategy was to treat mice topically with our repositioned drug set during the second and third OVA sensitization periods as previously described (Sun et al., 2011) (Fig. 6A).

A pilot study was first performed to test the toxicity (assessed by body weight change and induction of skin edema) of the repositioned drug set following topical administration to tape-stripped skin at 0.5, 2 and 10 mg/ml doses (see supplementary material Fig. S2A). No drugs (at the tested doses) produced a greater than 10% drop in body weight over the first 24 hours of treatment (see supplementary material Fig. S3). Of note, similar to phosphate-buffered saline (PBS)-treated controls, all drug-treated mice returned to approximately their pre-treatment weights over the 7-day treatment period (see supplementary material Fig. S3). When assessing skin edema, slight to moderate edema was observed at the treatment site with the 2 and 10 mg/ml doses for some drugs (see supplementary material Fig. S2B). In light of these results, we proceeded with a 0.5 mg/ml topical dose.

The severity of dermatitis was assessed by using the following quantifiable parameters, as previously described (Sun et al., 2011). A clinical score was quantified as the sum of individual grade scores, from 0 (no symptoms) to 3 (severe) for each of the following five symptoms: (1) erythema/hemorrhage; (2) edema; (3) excoriation/erosion; (4) scaling/drying; and (5) lichenification. A general histological score was quantified as the sum of individual grade scores, from 0 (no symptoms) to 3 (severe) for hypertrophy, hyperkeratosis and assessment of infiltrating inflammatory cells within the dermis and hypodermis. Specific counts for individual leukocyte subsets (neutrophils, mast cells, mononuclear cells and eosinophils) was also measured, expressed as the mean number of cells per ×400 field of view. Finally, skin myeoperoxidase (MPO) activity and serum OVA-specific IgE and IgG were measured by ELIZA. The synthetic corticosteroid triamcinolone acetonide was used as a positive control (Sun et al., 2011). When compared with PBS-treated negative controls, the effect of the repositioned drug set was scored for significance using both the Dunnett’s *t*-test and least significant difference (LSD) tests (Fig. 6B,C, and see supplementary material Fig. S4, Table S1 and Table S2). This analysis revealed that all drugs significantly suppressed at least one measured inflammatory parameter (Fig. 6C). Similar to the analysis of live neutrophil migration in our zebrafish system, the effects of the repositioned drug set on the severity of dermatitis appeared to cluster into two distinct groups. Pinacidil, nefopam hydrochloride, chlorphensin carbamate, acetohexamide and methyldopa (L,-) had moderate to little effect, whereas fludrocortisone acetate, amodiaquin dihydrochloride, alfuzosin hydrochloride, clonidine hydrochloride and mafenide hydrochloride had a more significant effect, in particular with respect to the clinical score, histological score and neutrophil quantification (Fig. 6C).

Collectively, these results confirm that at least a subset of the repositioned drug set can significantly suppress inflammation associated with this mouse model of AD, supporting the translation of these drugs as potential anti-inflammatories for therapeutic use.

## DISCUSSION

Here, we employed an *in vivo* phenotypic screen using the zebrafish system to identify previously unknown anti-inflammatory activities for existing drugs. Using a simple measurable readout of the inflammatory response (quantifying numbers of neutrophils at tail fin wounds), we identified a large number of drugs that diminished neutrophil recruitment, most of which have not previously been described as modulators of the inflammatory response. Ranking hits by potency of effect, and the removal of drugs that are known general anti-inflammatories, enabled us to select a manageable set of ten drugs for further analysis (our ‘repositioned drug set’). Live imaging of the effect of these drugs on neutrophil migration kinetics revealed that most drugs diminished neutrophil recruitment to wounds by suppressing migration velocity. Assessing the anti-inflammatory activities of the repositioned drugs in an ovalbumin-mediated epicutaneous mouse model of AD revealed several drugs as new potential therapeutic agents to treat AD. To our knowledge, this study represents the first drug-repositioning-focused screen employing the zebrafish system to expedite the repositioning of known drugs for inflammatory disease.

The best method to investigate the potential of compounds to interfere with complex physiological processes is to assess their effects *in vivo*. The zebrafish is ideally suited to *in vivo* phenotypic drug screening. High-throughput behavioral profiling using larval zebrafish has revealed new drugs that interfere with multiple complex behaviors (Rihel et al., 2010). Not only did this screen reveal potentially new psychotropic uses for drugs but, through hierarchical clustering of molecules to shared behavioral phenotypes, new pathways controlling rest or wake behaviors were also revealed. Owing to the inherent complexity of the inflammatory response, identifying new therapeutics for inflammatory conditions is especially suited to phenotypic screening (Saporito et al., 2012). Through phenotypic screening, the peroxisome proliferator-activated receptor-γ (PPAR-γ) agonist pioglitazone (Actos), which was originally approved for use as a diabetic therapeutic, has been shown to have therapeutic use in treating inflammatory bowel disease (Wang and DuBois, 2010). The initial selection criteria we used for anti-inflammatory activity was suppression of neutrophil recruitment to zebrafish tail fin wounds. Neutrophil migration to sites of inflammation is orchestrated by a range of signaling pathways that cooperate to manage the timely recruitment of neutrophils (Sanz and Kubes, 2012). Given such complexity, we anticipated that our phenotypic screening approach would likely reveal pharmacological agents that differ in both chemical structure and mechanism of action. From our zebrafish screen we identified 251 drugs that demonstrated a significant anti-inflammatory effect (*P*<0.05). This represented 22.4% of drugs in the library. We believe that this high hit rate is a direct reflection of the complexity of biological processes, collectively orchestrating neutrophil migration, that are potentially amenable to pharmacological interference. Despite this large number of hits, our screen design allowed us to effectively rank drug anti-inflammatory activity, as measured by their effect on neutrophil recruitment. Following removal of known anti-inflammatory drugs, we identified ten potent inhibitors of wound-induced neutrophil recruitment that had not previously been characterized as general anti-inflammatories. They were: chlorphensin carbamate, a muscle relaxant (blocks nerve impulses); fludrocortisone acetate, a mineralocoid used in the treatment of Addison’s disease (causes kidneys to retain sodium); acetohexamide, an antidiabetic medication (blocks ATP-sensitive K^+^ channels); methyldopa (L,-), an antihypertensive (L-aromatic amino acid decarboxylase inhibitor); pinacidil, a vasodilator (Ca^2+^-dependent K^+^ channel activator); amodiaquin dihydrochloride, an antimalarial (heme polymerase inhibitor); alfuzosin hydrochloride, an antihypertensive [α_1_-adrenergic receptor (AR) antagonist]; mafenide hydrochloride, an antibacterial (inhibitor of folic acid biosynthesis); nefopam hydrochloride, an analgesic (unknown mechanism of action); and clonidine hydrochloride, an antihypertensive (α_2_-AR agonist).

When categorized by therapeutic use, the largest groups of hits were known anti-inflammatories belonging to the NSAID and corticosteroid classes, vasodilator/antihypertensive drugs and antibacterials. Of note, 43.9% and 51.9% of all NSAIDs and corticosteroids that were present in the library, respectively, were identified as having conserved anti-inflammatory activity in our zebrafish screen. This proof-of-concept result suggests that core signaling pathways regulating inflammation are conserved in zebrafish, thus validating the zebrafish system as a discovery platform in which to pharmacologically interrogate the inflammatory response. Of all vasodilating/antihypertensive drugs represented in the library, 30.3% diminished neutrophil numbers in our tail fin wounding assay. Four of the drugs in the repositioned drug set fall into this therapeutic group. Two of these, alfuzosin hydrochloride (an α_1_-AR antagonist) and clonidine hydrochloride (an α_2_-AR agonist) are neuroendocrine modulators. It is well known that the inflammatory response is, in part, regulated by the sympathetic nervous system (Padro and Sanders, 2014). Repositioning of adrenergic agents has been suggested as a strategy to modulate inflammation associated with the neurodegenerative disease multiple sclerosis (Cosentino and Marino, 2013). Myeloid and lymphoid cell lineages express ARs on their cell surfaces that help regulate hematopoiesis, immune cell homing and functionally regulate immune cell activity in response to the sympathetic neurotransmitter norepinephrine (NE) (Padro and Sanders, 2014). These effects can often be contrasting, and depend on the level of NE and the type of adrenergic receptor stimulated. For example, in macrophages, exposure to the β_2_-AR agonist suppresses production of the pro-inflammatory cytokine TNF-α (Hu et al., 1991; Deng et al., 2004). This in contrast to α_1_- and α_2_-AR agonists, which elevate TNF-α production (Heijnen et al., 1996; Szelényi et al., 2000). With respect to monocytes/macrophages, it is generally believed that pro-inflammatory activity is inhibited by β_2_-AR, but enhanced by α_1_-AR or α_2_-AR, stimulation (Padro and Sanders, 2014). In neutrophils, β_2_-AR agonists have been shown to inhibit neutrophil activation (Johnson, 2002), whereas lipopolysaccharide (LPS)-induced mobilization of neutrophils from the bone marrow is dependent on α_1_-AR activity (Altenburg et al., 1997). Furthermore, in an animal model of epinephrine-driven stress, more neutrophils traffic to wounded tissue in a process dependent on interleukin-6 (IL-6) (Kim et al., 2014). Of note, through an unknown mechanism, clonidine (one of our repositioned drugs) has been shown to inhibit systemic IL-1β and IL-6 levels following ischemia-reperfusion in humans (Gourdin et al., 2012). The next largest therapeutic group for drugs identified from our screen was antibacterials (10.8% of all hits). It is emerging that, in addition to antibacterial activity, many macrolides, sulfonamides and tetracyclines affect innate immune cell activity, including migration, phagocytosis and pro-inflammatory cytokine production (Kwiatkowska et al., 2013). Our repositioned drug set included the sulfonamide-type antibacterial mafenide hydrochloride.

We decided to investigate conservation of activity of our repositioned drug set in an *in vivo* mouse model of AD for the following reasons. Persistent inflammation associated with inflammatory skin diseases (such as AD and psoriasis) causes considerable damage to the skin (Kupper, 1990; Boguniewicz and Leung, 2011). Strategies to treat these diseases largely rely on the immunosuppressive effects of corticosteroids. In addition to beneficial effects, including limiting the recruitment and/or activation of immune cells (such as neutrophils), corticosteroids can negatively impact the ability of the skin to repair (Guillot, 2013). Newer anti-inflammatory, anti-itch and antimicrobial agents are currently in clinical trials as potential medications for AD (Ong, 2012). However, there still exists an unmet need for newer therapeutic agents for symptomatic management of AD. Any one of our repositioned drugs that exhibited therapeutic efficacy in alleviating inflammation in this model has the potential for future use in treating AD. All drugs in our repositioned drug set demonstrated a significant reduction in at least one of the parameters used to measure inflammation in this model of AD, when compared with PBS-treated controls. As expected, fludrocortisone acetate [which has previously been shown to have therapeutic effect in treating allergic dermatitis (Vollmer, 1956)] was as effective in suppressing most measurements of inflammation as the positive control triamcinolone acetonide (a synthetic corticosteroid used to treat a number of inflammatory skin conditions). Of the repositioned drug set (not considering fludrocortisone acetate), amodiaquin dihydrochloride, alfuzosin hydrochloride, clonidine hydrochloride and mafenide hydrochloride were the most effective in alleviating inflammation, when compared with PBS-treated controls. In order of therapeutic effect, amodiaquin dihydrochloride, alfuzosin hydrochloride and clonidine hydrochloride demonstrated significant reductions when measuring clinical score, histological score and numbers of recruited neutrophils and mast cells, when compared with PBS-treated controls, using both the Dunnett’s *t*-test and LSD test to evaluate statistical significance. Mafedine hydrochloride treatment also resulted in significant reductions in these measurements when using the LSD test to determine significance. Further work is required to evaluate the impact of these drugs in other mammalian models of cutaneous inflammation and in alleviating skin inflammation in humans.

In summary, here we highlight the utility of the zebrafish to expedite identifying unknown anti-inflammatory activities for existing drugs. Employing a simple tail fin wounding assay, we identified a number of drugs with anti-inflammatory activity that had not previously been characterized as general anti-inflammatory agents. Using an ovalbumin-mediated epicutaneous mouse model of AD, we reveal that several repositioned drugs might have therapeutic use in the treatment of AD. This study provides compelling evidence that exploiting the zebrafish as an *in vivo* drug screening platform holds promise as a strategy to expedite the repositioning of existing drugs for new therapeutic uses.

## MATERIALS AND METHODS

### Zebrafish maintenance

Zebrafish (*Danio rerio*) embryos were obtained from natural spawnings and raised at 28°C in E3 medium supplemented with 0.003% phenylthiourea (PTU) to inhibit pigmentation. The neutrophil-specific *Tg(lyz:EGFP)^NZ117^* transgenic reporter line was used in this study. Research was conducted with approval from the University of Auckland Animal Ethics Committee.

### Mice

Six- to 8-week-old female C57BL/6 mice were bred and housed in the Animal Resource Unit, Faculty of Medical and Health Sciences, University of Auckland, Auckland, New Zealand. The mice were kept in an air-conditioned room with controlled humidity, temperature and a 12-hour light/dark cycle. All experiments were conducted under a protocol approved by the University of Auckland Animal Ethics Committee.

### Chemicals

The Prestwick Chemical Library^®^ (Prestwick Chemical), containing 1280 approved drugs, was used (http://www.prestwickchemical.com/index.php?pa=26). For larger quantities of the repositioned drug set, 10 mg of individual drug was obtained from Prestwick Chemical.

### Zebrafish chemical genetic screen

Three-dpf *Tg(lyz:EGFP)* larvae were transferred into screening medium [E3 medium supplemented with 1% DMSO, 20 μΜ metronidazole, 0.05 U/ml penicillin, 50 ng/ml streptomycin and 1 mM Tris (pH 7.4) (Murphey et al., 2006)] and arrayed into 48-well tissue-culture plates (5–7 larvae/well) using a fire-polished glass capillary to avoid unintentional damage to larvae. Two negative (1% DMSO-treated) and two positive [50 μΜ SP600125-treated (Calbiochem, cat# 420119)] controls we also included per plate. Larvae were exposed (by immersion) to a 1-hour drug pre-treatment at 1.25 μg/ml, prior to removal from drug and tail fin amputation within screening medium supplemented with tricaine (0.168 mg/ml). All transfers of larvae employed fire-polished glass capillaries to ensure no unintentional damage to larvae that could induce a secondary neutrophil response. Care was taken to transect tail fins at exactly the same anatomical location to ensure a consistent inflammatory response (see Fig. 1B,C). Following wounding, larvae were immediately returned to drug (at 1.25 μg/ml) for 4 hours followed by fixation in 4% paraformaldehyde (in PBS) and subsequent immunofluorescence detection of GFP and quantification of wound-responding neutrophils.

### Confocal imaging

Live embryos and larvae were mounted for confocal imaging as previously described (Hall et al., 2009a). All live confocal imaging was performed on an Olympus FV1000 FluoView laser scanning confocal microscope equipped with an incubation chamber. For live imaging of drug-treated larvae, larvae were immobilized for time-lapse imaging in 0.75% low-melting-point agarose in E3 medium supplemented with 0.003% PTU, 0.126 mg/ml tricaine and drug (1.25 μg/ml). Of note, when mounting and live-imaging in the presence of SP600125, a reduced dose of 25 μM was necessary owing to the development of cardiac edemas/labored circulation in some larvae when used at the screening concentration of 50 μM.

### Measuring migratory kinetics of neutrophils towards tail fin wounds

Neutrophil migratory paths were collected from DMSO-treated and drug-treated 3-dpf *Tg(lyz:EGFP)* larvae following tail transection. When generating wounds for live tracking experiments, smaller wounds (resulting from fin amputations half way between the posterior-most extremity of the notochord and the posterior fin edge) were generated. This was to create a smaller neutrophil response, making it easier to track individual neutrophils. Confocal time-lapse datasets were generated from two individual larvae/treatment using the following parameters: 320×320 pixels; ~7 μm Z-stacks through the entire trunk; 20× objective; 1.0× zoom. Z-stacks were collected every 90 seconds starting from ~45 minutes post-wounding. Similar imaging parameters were used, including laser voltage, gain, offset and scanning speed, to ensure any differences were not an artifact of altered image acquisition settings. Tracks were manually generated using Volocity 6.1.1 (Perkin Elmer), only tracks lasting longer than 15 minutes were used to measure the following parameters: track velocity, average speed over the whole track (μm/s); displacement rate, straight line distance from the first position in the track to the last divided by tracked time (μm/s); meandering index, the displacement rate divided by velocity, which provides a measure of a track’s deviation from a straight line – a value of 1 indicates that the track is a perfect straight line, and the smaller the value of the meandering index, the greater the meandering of the track.

### Flow cytometry

Flow cytometry was performed as previously described (Hall et al., 2009b).

### Immunofluorescence

Immunofluorescence detection of EGFP was performed with the following primary and secondary antibodies: chicken anti-GFP (1:500; Abcam, ab13970) with goat anti-chicken Alexa Fluor 488 (1:500; Invitrogen), as previously described (Hall et al., 2013).

### Ovalbumin-mediated epicutaneous sensitization model of AD

The methodology for inducing AD was as previously described (Sun et al., 2011). In brief, mice were shaved, and the dorsal skin tape-stripped six times under anesthesia. Ovalbumin (OVA; 100 mg in 100 ml of PBS, Sigma, St Louis, MO) was placed on a patch of gauze (1×1 cm), which was secured to the skin site with a transparent bio-occlusive dressing for a 1-week period and then removed. Two weeks later, mice were randomly assigned to treatment groups (six mice per treatment). Each mouse received a total of three 1-week exposures to a patch separated by 2-week patch-free intervals. For the second and third sensitizations, patches were soaked in 100 ml of PBS containing 100 mg OVA and 50 μg of the synthetic corticosteroid triamcinolone acetonide or 50 μg of the test drugs. The mice were monitored daily to ensure that the patches remained in place throughout the sensitization period. They were sacrificed 24 hours after removal of the third patch, and blood and skin tissue samples were collected. Serum was separated from blood samples, and stored at −80°C.

### Assessment of dermatitis

The assessment of the severity of dermatitis was performed on completion of the third sensitization, and was expressed as the sum of the individual score grades of 0 (no symptoms), 1 (mild), 2 (moderate) or 3 (severe) for each of the following five symptoms: (1) erythema/hemorrhage, (2) edema, (3) excoriation/erosion, (4) scaling/drying and (5) lichenification (Sun et al., 2011).

### Scoring of skin pathology by histological analysis

Skin tissues were fixed in 10% formalin, embedded into paraffin, sectioned, stained with hematoxylineosin, and examined by light microscopy in a blinded fashion. The dermis was assessed for hypertrophy and hyperkeratosis, and both the dermis and hypodermis were examined for infiltration by inflammatory cells. The overall histology score was expressed as the sum of the individual score grades according to the following scoring system: 0 (no symptoms), 1 (mild), 2 (moderate) or 3 (severe) (Sun et al., 2011).

### Enumerating infiltrating inflammatory cells

Skin sections were examined for infiltration by neutrophils and mononuclear cells. Eosinophils were identified in sections after staining with hematoxylin-Biebrich scarlet, and mast cells after staining with toluidine blue. The numbers of neutrophils, eosinophils, mononuclear cells and mast cells were counted blindly in 20 high-power field views (at ×400) and expressed as the mean number of cells per field of view.

### Enzyme-linked immunosorbent assay

The methodology for measuring OVA-specific IgE and IgG1 has been described previously (Kanwar et al., 2008).

### Tissue myeloperoxidase activity assay

Skin myeloperoxidase (MPO) activity, an indicator of polymorphonuclear leukocyte infiltration, was assessed using a mouse MPO ELISA kit (HK210; Hycult Biotech Inc., Suite, PA) as previously described (Otuki et al., 2011).

### Statistical analysis

Statistical analyses were performed using Prism 5.0 (GraphPad Software, Inc.). For the zebrafish screen statistical significance was assessed using unpaired, two-tailed *t*-tests. An average *P*-value was calculated by comparing the effect of a specific drug against the 2 DMSO controls on the same plate. The effect of drugs on neutrophil recruitment was then ranked based on the resulting *P*-value. All data presented in scatter plots show means ± s.d. For the mouse dermatitis study, results were expressed as mean ± s.d., and a one-way analysis of variance (ANOVA) followed by a Dunnett’s *t*-test and LSD test to evaluate statistical significance. One-way ANOVA was used by including all the groups except the healthy control. *P*-values <0.05 were considered statistically significant.

## Supplementary Material

Supplementary Material
