# Supplementary material for: Repositioning drugs for inflammatory disease – fishing for new anti-inflammatory agents
Source: Dis Model Mech. 2014 Jul 18;7(9):1069–81. doi: 10.1242/dmm.016873 (PMC4142727; doi:10.1242/dmm.016873)
Supplement: Supplementary Material [file supp_7_9_1069__index.html]

Repositioning drugs for inflammatory disease – fishing for new anti-inflammatory agents — Supplementary Material 

# Repositioning drugs for inflammatory disease – fishing for new anti-inflammatory agents

## DMM016873 Supplementary Material

**Files in this Data Supplement:**

- **Supplementary Material**
