# Supplementary material for: Repositioning drugs for inflammatory disease – fishing for new anti-inflammatory agents
Source: Dis Model Mech. 2014 Jul 18;7(9):1069–81. doi: 10.1242/dmm.016873 (PMC4142727; doi:10.1242/dmm.016873)
Supplement: Supplementary Material [file supp_7.9.1069_DMM016873.pdf]

Supplementary material

Repositioned drug set has no significant effect on whole-larva neutrophil numbers, relative to DMSO controls.

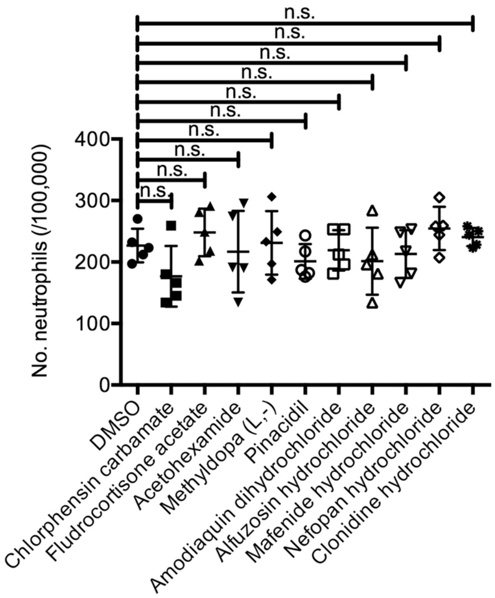

Figure S1. Flow cytometry quantification of whole-larva neutrophil numbers (measured as numbers of fluorescent neutrophils/100,000 cells) from dissociated 3 dpf *Tg(lyz:EGFP)* larvae following indicated drug treatments (data displayed as mean ± S.D., n=5 groups of ~50 larvae/treatment). Abbreviation: n.s., not significant.

Topical administration of repositioned drug set has no toxic effect on skin when administered at 0.5 mg/ml.

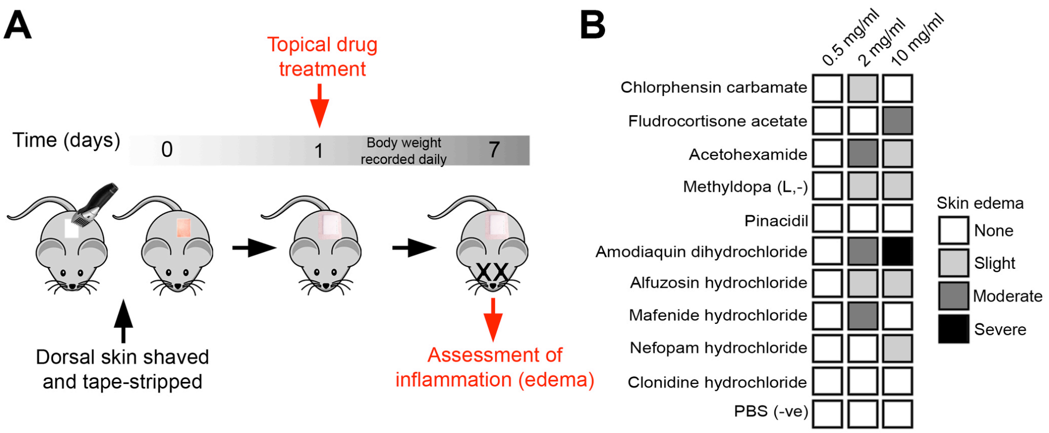

Figure S2. (A) Schematic illustrating design of pilot study to test toxicity of selected drugs delivered by topical administration. Shaved exposed skin is tape-stripped prior to topical delivery of sterile drug solutions (0.5, 2 and 10 mg/ml, n=4 mice/dose) on a patch of sterile gauze secured with bio-occlusive dressing. Inflammation is visually assessed following 1 week. (B) Summary of drug toxicity (as assessed by visual inspection of skin for edema) at 0.5, 2 and 10 mg/ml doses. The toxicity of drugs is presented as a grey-scale with lighter shades representing less/or no edema, and darker shades more severe edema.

Topical administration of repositioned drug set has no significant effect on body weight, relative to PBS controls.

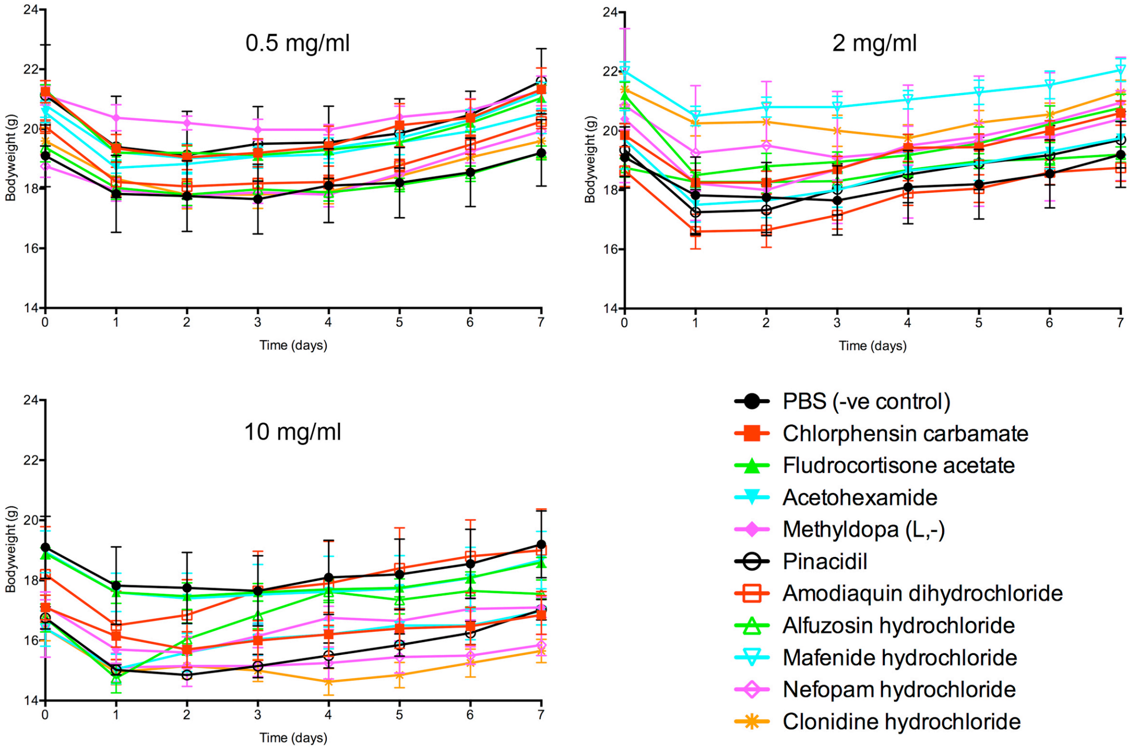

Figure S3. Bodyweights of mice during pilot study to help assess toxicity of repositioned drug set following topically delivery (at 0.5, 2 and 10 mg/ml doses). Weights are displayed as mean ± S.D. (n=4 mice/group).

Graphs of inflammatory parameters examined in ovalbumin-mediated epicutaneous sensitization model of AD following treatment with repositioned drug set.

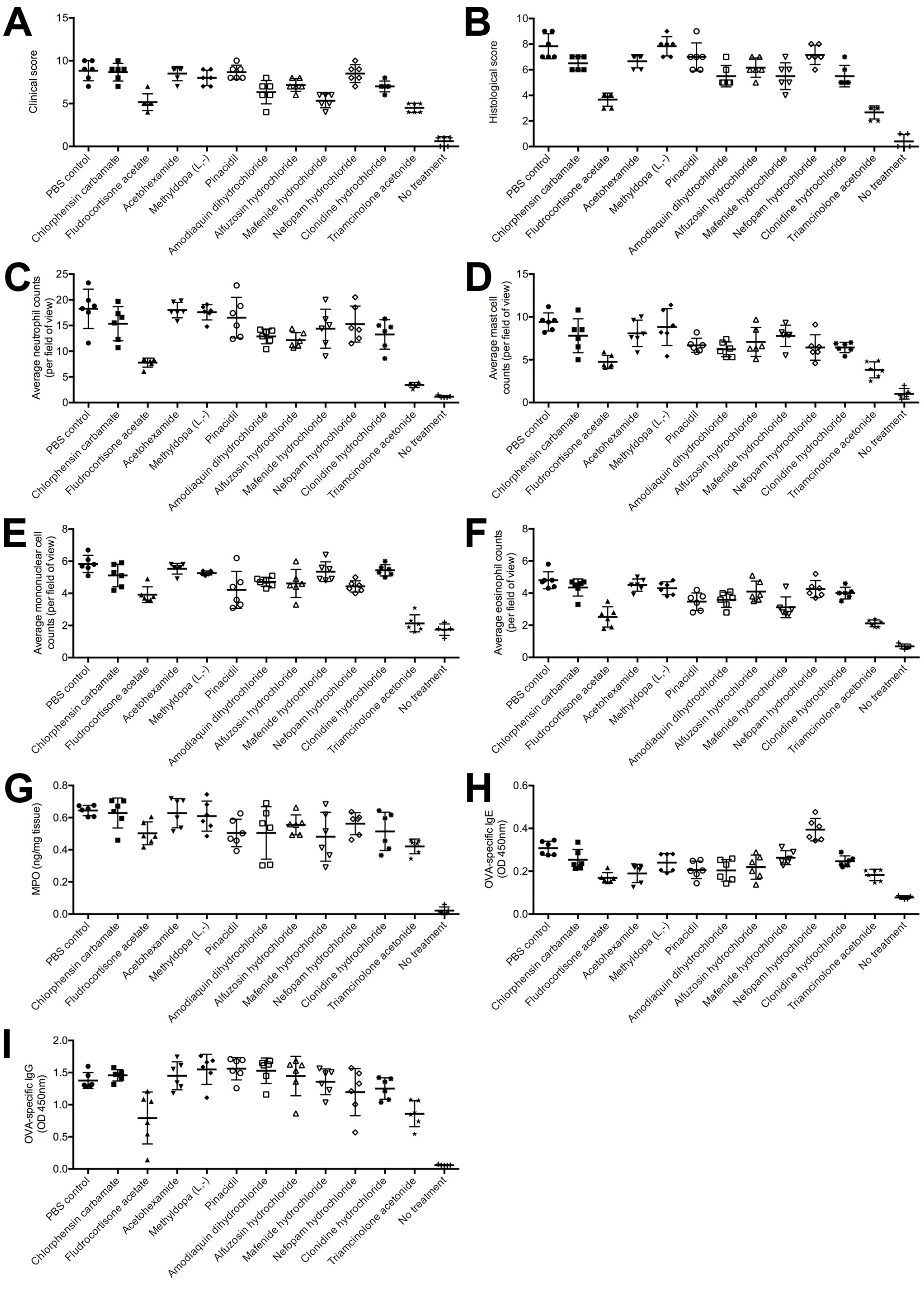

Figure S4. Effects of selected drugs on (A) clinical score (visual assessment), (B) histological score (examination of dermis and hypodermis from haematoxylin–eosin-

stained skin sections), (C-F) histological quantification of neutrophils, mast cells, mononuclear cells and eosinophils, respectively, and ELISA quantification of MPO (G) and OVA-specific IgE (H) and IgG (I). Data displayed as mean ± S.D. (n=6 mice/group).

Table S1. Statistical analysis of inflammatory parameters examined in ovalbumin-mediated epicutaneous sensitization model of AD following treatment with the repositioned drug set using the Post hoc Dunnet-t test, relative to PBS control treatment (p-values<0.05 are highlighted in red).

*Asterisk marks a significant increase in an inflammation parameter, relative to PBS controls.

Drug	Clinical score	Histological score	Neutrophils	Mast cells	Mononuclear cells	Eosinophils	MPO	IgE	IgG
**Chlorphensin carbamate**	0.99999937	0.03884138	0.35145440	0.24806996	0.24524483	0.59333749	0.99999979	0.15999814	0.99909391
**Fludrocortisone acetate**	0.00000006	0.00000003	0.00000005	0.00000071	0.00000375	0.00000003	0.09263091	0.00000105	0.00057467
**Acetohexamide**	0.99854732	0.09577988	1.00000000	0.46574174	0.97567950	0.92991757	0.99999958	0.00003124	0.99962647
**Methyldopa (L,-)**	0.59355154	1.00000000	0.99995405	0.99028633	0.51352449	0.46612564	0.99835051	0.03917486	0.80501689
**Pinacidil**	0.99999937	0.40564698	0.88896728	0.00626985	0.00010627	0.00017283	0.10132090	0.00042788	0.75339880
**Amodiaquin dihydrochloride**	0.00014064	0.00002920	0.00668962	0.00087270	0.01401893	0.00071932	0.10116353	0.00029259	0.89439343
**Alfuzosin hydrochloride**	0.02243422	0.00472849	0.00142424	0.02834591	0.00580818	0.12718695	0.55065361	0.00276245	0.99979203
**Mafenide hydrochloride**	0.00000013	0.00002920	0.09712738	0.23798770	0.69802901	0.00000174	0.03490174	0.34437879	1.00000000
**Nefopam hydrochloride**	0.99854732	0.67072434	0.32579861	0.00223578	0.00100904	0.35304318	0.65400712	0.00369128*	0.76635849
**Clonidine hydrochloride**	0.00910965	0.00002920	0.01418774	0.00223578	0.86381070	0.05606950	0.14921280	0.07788345	0.96684728
**Triamcinolone acetonide**	0.00000003	0.00000003	0.00000003	0.00000003	0.00000003	0.00000003	0.00129649	0.00000966	0.00294007
**Healthy**	0.00000003	0.00000003	0.00000003	0.00000003	0.00000003	0.00000003	0.00000003	0.00000003	0.00000003

Table S2. Statistical analysis of inflammatory parameters examined in ovalbumin-mediated epicutaneous sensitization model of AD following treatment with the repositioned drug set using the Post hoc LSD test, relative to PBS control treatment (p-values<0.05 are highlighted in red).

*Asterisk marks a significant increase in an inflammation parameter, relative to PBS controls.

Drug	Clinical score	Histological score	Neutrophils	Mast cells	Mononuclear cells	Eosinophils	MPO	IgE	IgG
**Chlorphensin carbamate**	0.75330542	0.00442392	0.05779878	0.03726129	0.03673995	0.11938838	0.78200112	0.02197669	0.55079551
**Fludrocortisone acetate**	0.00000000	0.00000000	0.00000000	0.00000006	0.00000032	0.00000000	0.01166917	0.00000009	0.00005289
**Acetohexamide**	0.53011025	0.01212242	0.95590116	0.08414709	0.37519806	0.29661332	0.76810112	0.00000274	0.58662025
**Methyldopa (L,-)**	0.11945406	1.00000000	0.65845413	0.43274445	0.09651188	0.08424296	0.52433393	0.00446560	0.20276593
**Pinacidil**	0.75330542	0.06977190	0.25704576	0.00062810	0.00000947	0.00001552	0.01292710	0.00003911	0.17784073
**Amodiaquin dihydrochloride**	0.00001258	0.00000256	0.00067237	0.00008119	0.00147138	0.00006658	0.01290412	0.00002653	0.26154506
**Alfuzosin hydrochloride**	0.00243587	0.00046730	0.00013443	0.00313733	0.00057964	0.01679991	0.10677479	0.00026687	0.60837481
**Mafenide hydrochloride**	0.00000001	0.00000256	0.01231727	0.03541022	0.15509791	0.00000015	0.00393519	0.05629960	0.88381699
**Nefopam hydrochloride**	0.53011025	0.14500207	0.05243033	0.00021428	0.00009426	0.05813739	0.13912101	0.00036082*	0.18370245
**Clonidine hydrochloride**	0.00093108	0.00000256	0.00149028	0.00021428	0.23817741	0.00663471	0.02024328	0.00958662	0.35373422
**Triamcinolone acetonide**	0.00000000	0.00000000	0.00000000	0.00000000	0.00000000	0.00000000	0.00012201	0.00000084	0.00028472
**Healthy**	0.00000000	0.00000000	0.00000000	0.00000000	0.00000000	0.00000000	0.00000000	0.00000000	0.00000000
